# Clinical Characteristics and Management of Four Cases of Visceral Leishmaniasis-Associated Hemophagocytic Lymphohistiocytosis

**DOI:** 10.4269/ajtmh.25-0530

**Published:** 2026-03-26

**Authors:** Meng Tian, Jing Li, Shuzhi Dai, Lijuan Ma

**Affiliations:** Department of Clinical Laboratory, Capital Center for Children’s Health, Capital Medical University, Capital Institute of Pediatrics, Beijing, China

## Abstract

The aim for the present study was to analyze clinical features, diagnostic approaches, and therapeutic strategies for visceral leishmaniasis (VL)-associated hemophagocytic lymphohistiocytosis (HLH) in pediatric patients. The clinical characteristics and test results of the children were summarized. Among the four patients, three resided in VL-endemic regions, and one had traveled to a VL-endemic region. All patients presented with recurrent fever (>38.5°C), hepatosplenomegaly, and decreased hemoglobin (HGB) levels ([78.75 ± 8.50] g/L) and platelet (PLT) counts ([59.50 ± 17.48] × 10^9^/L). Before a definitive diagnosis could be made, patients exhibited progressive declines in white blood cell counts, HGB levels, and PLT counts, along with elevated triglyceride, serum cytokine (interleukin [IL]-6, IL-10, IL-2R, and tumor necrosis factor α) levels. Bone marrow aspirate smears revealed hemophagocytosis and *Leishmania donovani* (LD) bodies in all cases: two were diagnosed via direct identification of LD bodies, one was diagnosed through re-examination of bone marrow smears after confirming a travel history, and one was diagnosed via re-examination prompted by metagenomic next-generation sequencing, which revealed leishmaniasis. All the patients were initially diagnosed with HLH and received HLH-directed immunochemotherapy before VL diagnosis, with suboptimal response. After confirmation of VL, sodium stibogluconate therapy was initiated, resulting in a partial response in all cases. Etiological investigation is critical for HLH diagnosis. For VL-associated HLH, sodium stibogluconate as targeted therapy rapidly controls HLH, facilitates immunosuppression withdrawal, and significantly improves patient outcomes. White blood cell count, HGB level, PLT count, and lactate dehydrogenase level may serve as critical prognostic biomarkers for VL-associated HLH.

## INTRODUCTION

Hemophagocytic lymphohistiocytosis (HLH) is a severe hyperinflammatory syndrome that results from genetic or acquired immune dysregulation.^1^ It is classified as primary HLH, an autosomal or X-linked recessive disorder, and secondary HLH, which is triggered by underlying conditions such as infections, malignancies, or rheumatic diseases, with infections being the most common etiology.^2^^,^^3^ Secondary HLH typically occurs without a family history or known genetic defects. Its clinical presentation is characterized by persistent fever, hepatosplenomegaly, and hemophagocytosis. Hemophagocytic lymphohistiocytosis is rapidly progressive and highly fatal, with a median survival of less than 2 months if left untreated.^4^^,^^5^ Early identification of the underlying cause and targeted therapy are therefore critical for treatment efficacy and prognosis.

Visceral leishmaniasis (VL), also known as kala-azar, is an endemic parasitic disease caused by infection with *Leishmania donovani* (LD). The primary transmission route is through the bite of infected phlebotomine sandflies. When sandflies feed on infected hosts (typically humans or canines), they ingest the parasites, which then multiply within the insect’s gut. Subsequent bites inoculate promastigotes into new hosts, where they transform into amastigotes and proliferate within macrophages. Globally, VL affects ∼500,000 new cases annually, threatening 350 million people across 88 endemic countries spanning the Mediterranean, North/East Africa, the Middle East, Central/West Asia, the Indian subcontinent, and the Americas. In 2020, ∼80% of cases were reported from seven countries: Afghanistan, Algeria, Brazil, Colombia, Iraq, Pakistan, and Syria.^6^^–^^8^ Currently, endemic areas in China are primarily concentrated in Xinjiang, Gansu, Sichuan, Shaanxi, Shanxi, and Inner Mongolia.^9^ In recent years, driven by global climate change and socioeconomic development, historically eliminated VL foci in central and western China have experienced disease recrudescence. This resurgence is characterized by an expanding geographical spread and a significantly increasing incidence.^10^^,^^11^ In 2023, 18 newly identified endemic counties reported recrudescence, with 299 confirmed cases nationwide—representing a 25.1% increase compared with 2022 surveillance data.^12^^–^^15^ The clinical manifestations include prolonged irregular fever, chills, and hepatosplenomegaly. Hematological abnormalities (varying degrees of decreases in white blood cell [WBC] count, hemoglobin [HGB] levels, and platelet [PLT] counts) are present in most cases, with anemia being the most prevalent (>90%). The diagnostic gold standard remains microscopic identification of amastigotes (LD bodies) in Giemsa-stained samples from bone marrow, lymph nodes, or splenic aspirates, which exhibit morphological characteristics of pale blue cytoplasm with distinct purple nuclei and kinetoplasts. However, parasite load varies according to the sampling site and disease stage, and detection requires substantial expertise, contributing to low sensitivity. The nonspecific clinical presentation further increases the risk of misdiagnosis.

With VL control improving in endemic regions over the years, VL-associated HLH has become increasingly rare, leading to low clinical suspicion among physicians when evaluating HLH cases. The overlapping clinical features between VL and HLH often obscure the underlying parasitic infection. Gagnaire et al.^16^ reported an initial misdiagnosis in 30% (4/12) of VL-associated HLH cases. Sole reliance on HLH-directed immunochemotherapy without antiparasitic treatment frequently results in disease recurrence or progression, delaying appropriate care^17^ and posing further difficulties for diagnosis and treatment. The present retrospective analysis of four pediatric VL-associated HLH cases was conducted to improve clinicians’ diagnostic awareness and inform therapeutic decision-making for this condition.

## MATERIALS AND METHODS

### Patient data.

Four cases of VL-associated HLH diagnosed at the Capital Center for Children’s Health (Beijing, China) between May 2015 and November 2023 were retrospectively analyzed. The Capital Center for Children’s Health is a national children’s medical center with more than 2 million outpatient visits and 40,000 hospitalizations annually. Collected data included demographics (age and sex), epidemiological data (native region, travel history to endemic areas, and history of sandfly bites), clinical manifestations and vital signs (fever and hepatosplenomegaly), HLH-related laboratory tests (hematology, biochemistry, coagulation, ferritin, bone marrow cytology, and cytokine profile), genetic testing for primary HLH, etiological workup for VL (*Leishmania* IgG serology and bone marrow smear microscopy), and other pathogen testing (microbial cultures, pathogen-specific serology/nucleic acid tests, and metagenomic next-generation sequencing [mNGS]).

### Diagnostic criteria.

The diagnosis of VL involves an assessment of exposure history, clinical presentation, serological test results, and direct parasitological evidence. According to the “Diagnostic Criteria and Management Guidelines for Visceral Leishmaniasis” under the Law of the People’s Republic of China on the Prevention and Treatment of Infectious Diseases and its Implementation Measures, VL diagnosis requires: 1) residence in or travel to endemic regions, 2) relevant clinical symptoms, and 3) the identification of amastigotes (LD bodies) on a bone marrow smear. The diagnosis of HLH is confirmed on the basis of the HLH-2004 criteria, which may be met via either of the following options: 1) molecular confirmation: identification of known HLH-associated pathogenic genes, such as PRF1, UNC13D, STX11, STXBP2, Rab27a, LYST, SH2D1A, BIRC4, ITK, AP3β1, MAGT1, CD27 and other pathological mutations in HLH-associated genes, or 2) fulfillment of ≥5 of the eight clinical or laboratory criteria: 1) fever: >38.5°C for ≥7 days; 2) splenomegaly; 3) cytopenia (affecting ≥2 lineages): an HGB level <90 g/L (<100 g/L in neonates <4 weeks), a PLT count <100 × 10^9^/L, a neutrophil count <1.0 × 10^9^/L (non-marrow suppression causes); 4) hypertriglyceridemia (fasting triglycerides >3 mmol/L or >3 standard deviations above age-adjusted norms) or hypofibrinogenemia (<1.5 g/L or >3 standard deviations below age-adjusted norms); 5) hemophagocytosis in the bone marrow, spleen, liver, or lymph nodes; 6) reduced or absent natural killer (NK) cell activity; 7) hyperferritinemia (≥500 µg/L); and 8) an elevated soluble interleukin (IL)-2 receptor (sCD25) level.

### Test method.

#### Etiological examination.

Bone marrow aspirate smears were prepared and stained with Giemsa for microscopic identification of *Leishmania* amastigotes (LD bodies).

#### Immunological testing.

Anti-*Leishmania* IgG antibodies were detected using recombinant K39 (rK39)-based immunochromatographic rapid diagnostic tests.

#### Molecular biological testing.

Metagenomic next-generation sequencing was performed to identify pathogens.

### Efficacy evaluation.

The indicators for efficacy evaluation of HLH^18^ included serum sCD25 levels, ferritin levels, blood cell counts, triglyceride levels, hemophagocytosis, and neurological status (for central nervous system-HLH cases): 1) complete response (CR): all of the aforementioned parameters have returned to the normal range; 2) partial response (PR): ≥2 symptom or laboratory parameters exhibiting >25% improvement, with specific thresholds: 1) sCD25 reduction ≥1/3; 2) a ferritin and triglyceride reduction of ≥25%; 3) neutrophil recovery (without blood transfusion): a 100% increase to >0.5 × 10^9^/L for those with levels <0.5 × 10^9^/L, a 100% increase to normal range for those with levels of 0.5–2.0 × 10^9^/L; 4) alanine aminotransferase levels >400 U/L: a ≥50% reduction; 3) no response: failure to meet the abovementioned criteria. To ensure transparent evaluation of efficacy, the defined criteria for PR were consistently applied to all cases. Specifically, changes in serum sCD25 and ferritin levels were calculated on the basis of their absolute values before and after treatment, as outlined in Supplemental Table 1. These changes were confirmed by comparing baseline and post-treatment values.

## STATISTICAL ANALYSES

Data were sorted using Microsoft Excel software (Microsoft Corp., Redmond, WA). Given the small sample size (*n* = 4), the use of a paired *t*-test for statistical comparison was reassessed because the power of this test is extremely limited with such a small cohort, and the assumption of normality cannot be reliably validated. Consequently, descriptive statistics, including mean values, standard deviations, and ranges for continuous variables, were used alongside individual patient data. This methodology ensures transparency and mitigates the risk of overinterpreting statistical significance. Because of the limited sample size, *P*-values were presented solely for illustrative purposes and should be interpreted with caution; no inferential conclusions were drawn exclusively on the basis of these values. Furthermore, individual patient data were presented to provide a more precise representation of treatment outcomes.

## RESULTS

### General information.

The case series included three men (75%) and one woman (25%), with a median age at onset of 1.7 years (range: 1.3–2.1 years). Initial admissions were made to the hematology department (*n* = 3) and the rheumatology department (*n* = 1).

### Epidemiological investigation.

All patients had a history of exposure to *Leishmania*-endemic regions, including three cases (75%) from Shanxi Province (two from Taiyuan City, one from Yangquan City) and one case (25%) with a travel history to Hebei Province (Jingxing County and Zhangjiakou City). Only one patient (25%) reported definitive sandfly bites.

### Clinical symptoms.

All patients presented with irregular recurrent fever (>38.5°C), with intervals ranging from 3–4 hours to 7–8 hours. Transient defervescence was achieved after antibiotics or symptomatic treatment, followed by unexplained recurrence. Hepatosplenomegaly was identified in all four cases, pallor or anemic appearance was observed in three cases, and bilateral inguinal petechial rash was present in one case.

### Laboratory tests.

**Figure 1.**
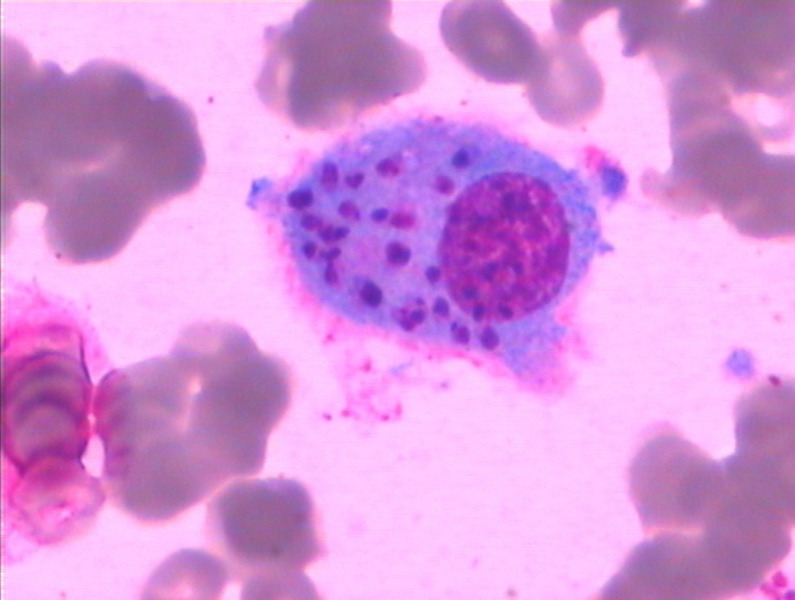
Phagocytosed *Leishmania donovani* bodies within macrophages in a bone marrow smear from Case A (Wright-Giemsa staining × 1,000).

**Figure 2.**
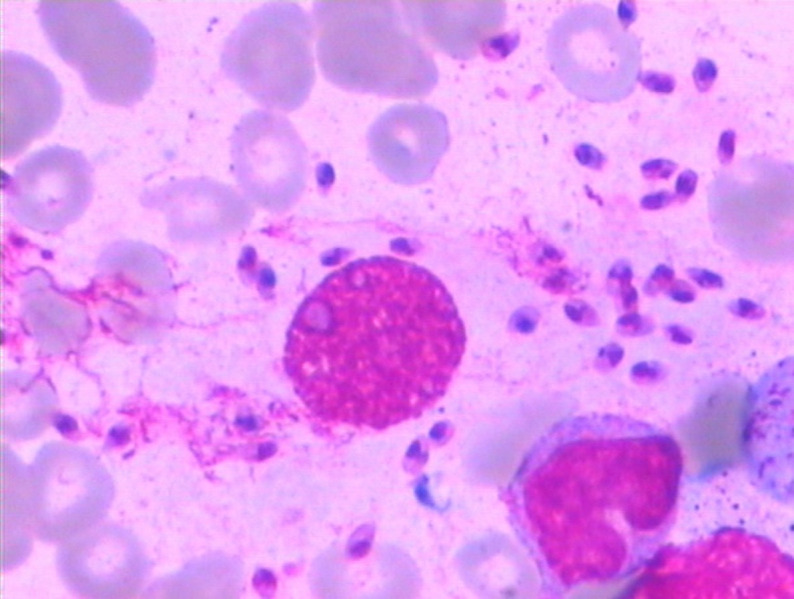
Scattered extracellular *Leishmania donovani* bodies in a bone marrow smear from Case A (Wright-Giemsa staining × 1,000).

Upon initial consultation in the hematology department, four patients (denoted as A, B, C, and D) had WBC counts ([2.50 ± 1.14] × 10^9^/L), HGB levels ([78.75 ± 8.50] g/L), and PLT counts ([59.50 ± 17.48] × 10^9^/L) all below the reference range. Serum ferritin levels were elevated in all cases, and lactate dehydrogenase (LDH) levels were significantly elevated. Moreover, three patients exhibited neutropenia, and three had elevated triglycerides (Table 1). Initial cytokine profiling revealed significantly elevated levels of IL-6, IL-10, IL-2R, and tumor necrosis factor α in all patients (Table 2). A bone marrow aspiration and morphological examination revealed hemophagocytosis in all four patients; however, no LD bodies were initially identified. Diagnostic confirmation varied among cases: Case A was confirmed after repeated history-taking revealed exposure to endemic regions, followed by a second bone marrow aspiration that revealed LD bodies (Figures 1 and 2). Cases B and C were diagnosed through subsequent bone marrow aspirations that directly revealed LD bodies. Case D was confirmed when mNGS of blood samples revealed *Leishmania*, prompting reexamination of bone marrow smears, which then revealed LD bodies (Supplemental Figures 1 and 2). The time from initial presentation to definitive diagnosis ranged from 17 days (Case A) to 7 months and 26 days (Case D). Hospitalization durations varied correspondingly: Case A was hospitalized for 1 month and 11 days compared with Case D’s hospitalization of 8 months and 22 days. Serological testing revealed positive *Leishmania* IgG antibodies in three patients (Cases A, C, and D), whereas Case B tested negative. Additionally, Case D had leishmaniasis, as confirmed via mNGS.

**Table 1:** Initial hematologic parameters of the four patients during their first hematology consultations

Case No.	WBC (10^9^/L)	ANC (10^9^/L)	HGB (10^9^/L)	PLT (10^9^/L)	sCD25 (U/L)	Ferritin (ng/mL)	Triglyceride (mmol/L)	LDH (U/L)	HDBH (U/L)	FIB (g/L)	NK cell activity (%)
A	3.00 ⬇	0.47 ⬇	73 ⬇	56 ⬇	–	>2,000 ⬆	2.52 ⬆	864 ⬆	660 ⬆	1.44	23 ⬆
B	2.24 ⬇	0.21 ⬇	70 ⬇	46 ⬇	>44,000 ⬆	>2,000 ⬆	3.49 ⬆	1,463 ⬆	1,017 ⬆	0.92	15
C	3.73 ⬇	1.07	85 ⬇	51 ⬇	>44,000 ⬆	1,141.98 ⬆	3.55 ⬆	661 ⬆	483 ⬆	2.45	17.05
D	1.05 ⬇	0.37 ⬇	87 ⬇	85	15,316 ⬆	6,093.13 ⬆	1.56	702 ⬆	487 ⬆	4.29	12.09

⬆ = above normal range; ⬇ = below normal range; – = not tested; ANC = absolute neutrophil count; FIB = fibrinogen; HDBH = alpha-hydroxybutyrate dehydrogenase; HGB = hemoglobin; LDH = lactate dehydrogenase; NK = natural killer; PLT = platelet; WBC = white blood cell.

**Table 2:** Cytokine profiles of the four patients at initial presentation

Case No.	IL-6 (pg/mL)	IL-8 (pg/mL)	IL-10 (pg/mL)	IL-2R (U/mL)	IL-1β (pg/mL)	TNF-α (pg/mL)
A	37 ⬆	44.4	83.8 ⬆	> 7,500 ⬆	–	45.8 ⬆
B	791 ⬆	648 ⬆	148 ⬆	> 7,500 ⬆	64.7 ⬆	114 ⬆
C	119 ⬆	29.4 ⬆	44 ⬆	> 7,500 ⬆	25 ⬆	92.4 ⬆
D	151 ⬆	211	93.5 ⬆	2103 ⬆	5.81 ⬆	79.8 ⬆

⬆ = above normal range; – = not tested; IL = interleukin; TNF-α = tumor necrosis factor α.

### Treatment and outcome.

Before the diagnosis of VL, all patients received HLH-directed empirical treatments, including antibiotics, immunomodulators, and cytotoxic drugs. Although these interventions temporarily normalized body temperature, all patients experienced recurrent fever and progressive cytopenia, indicating treatment failure. Three patients (Cases A–C) were managed primarily by the hematology department but exhibited persistent fever and elevated inflammatory markers despite anti-infective therapy, dexamethasone for cytokine storm control, and even HLH-directed chemotherapy. Case D presented differently and was initially evaluated by rheumatology for suspected macrophage activation syndrome (MAS) secondary to rheumatic disease. A comprehensive workup, including genetic testing, ruled out monogenic autoinflammatory disorders, whereas pathogen detection and autoimmune markers were unremarkable. The absence of a characteristic rash, arthritis, or active rheumatologic disease excluded rheumatic causes, prompting a hematology referral. During a hematology consultation, Case D exhibited a WBC count of 1.05 × 10^9^/L, a neutrophil count of 0.37 × 10^9^/L, an HBG level of 87 g/L, a PLT count of 185 × 10^9^/L, hemophagocytosis in bone marrow aspirate smears, a serum ferritin level of >500 ng/mL, NK cell activity of 12.09%, and an sCD25 level of 15,316 U/L (>2,400 U/L), meeting seven of eight HLH diagnostic criteria, leading to a diagnosis of HLH. Initial rheumatologic management included methylprednisolone pulse therapy, cyclosporine (25 mg, May 2023), and tocilizumab, with transient improvement. However, MAS recurred after 1 month (June 2023), and intervention according to the HLH protocol was administered, resulting in fever resolution and improvement in relevant laboratory parameters. A second MAS episode in August 2023 prompted intravenous immunoglobulin (5 g daily for 3 days), followed by planned etoposide (VP16) infusions for 5–8 weeks. Because of persistent fever and inflammation despite these measures, the hematology department recommended interferon (IFN)-γ monoclonal antibody treatment (emapalumab, 1 mg/kg for two doses) combined with ganciclovir prophylaxis. After seven sessions of IFN-γ monoclonal antibody treatment, the patient remained febrile with fluctuating inflammatory markers, indicating a protracted relapsing course. In summary, all four pediatric cases exhibited persistent, fluctuating disease without improvement before targeted VL treatment.

After VL diagnosis, all patients discontinued chemotherapy and gradually tapered antibiotics and immunomodulators, such as glucocorticoids, and received sodium stibogluconate therapy (total dose 200–240 mg/kg, administered as 0.5 g daily via intramuscular injections over 6 days) according to the “Chinese Expert Consensus on the Diagnosis and Treatment of Leishmaniasis.” Cases A and D required two treatment courses, whereas Cases B and C responded to one course. Case B experienced antimonial-related fever and decreased neutrophil count after 3 days of treatment, necessitating a 2-day treatment interruption before successful completion. Treatment was well tolerated in the remaining three cases. All four patients achieved favorable clinical outcomes after receiving antimonial therapy. Among them, laboratory parameters, including WBC, HGB, PLT, and LDH levels, revealed statistically significant differences before and after treatment (*P* <0.05; Supplemental Table 1), indicating marked therapeutic efficacy. Post-therapeutic laboratory assessments revealed consistent normalization of WBC, ANC, and PLT levels across all four cases. For each parameter, such as sCD25 and ferritin levels, the degree of improvement was determined according to predefined PR thresholds, with a ≥1/3 reduction in sCD25 levels and a ≥25% reduction in ferritin levels. This analysis is shown in Supplemental Table 1, where the improvement for each parameter is highlighted for all patients. Notably, Case A exhibited a 35% reduction in sCD25 and a 30% reduction in ferritin, both of which surpassed the established thresholds for PR. Hemoglobin levels returned to normal ranges in three patients, whereas in the remaining patient, they increased by >25%. Lactate dehydrogenase values normalized in one patient and decreased by >25% in the other three patients (Supplemental Table 2). All pediatric patients achieved body temperature normalization and regression of hepatosplenomegaly to normal size. After antileishmanial therapy, HLH manifestations were alleviated in all cases, with efficacy evaluation confirming PR in all four patients. Notably, a post-treatment bone marrow evaluation in Case A confirmed the eradication of LD bodies (Supplemental Figures 3 and 4).

## DISCUSSION

Notably, VL was recognized by the WHO as a neglected tropical disease in 2015^19^ and continues to affect an estimated 50,000 to 90,000 individuals globally every year.^18^ In the present case series, although only one pediatric patient had a documented sandfly bite history, all four patients had traveled to VL-endemic regions. This underscores the imperative for clinicians to maintain updated knowledge of VL-endemic areas and maintain high diagnostic suspicion when evaluating patients from these regions.

The reported incidence of VL-associated HLH is ∼2.1%,^20^ with *Leishmania* species emerging as the most prevalent protozoan trigger of HLH.^21^ The diagnostic challenge lies in the substantial clinical overlap between VL and HLH, both presenting with recurrent fever, hepatosplenomegaly, and progressive pancytopenia. Bode et al.^22^ particularly emphasize the diagnostic challenges associated with HLH secondary to VL in pediatric populations. In the present case series, three cases were initially diagnosed as HLH, whereas one was suspected to be MAS (HLH secondary to rheumatic diseases), and VL was subsequently identified as the underlying etiology. According to the “Chinese Expert Consensus on the Diagnosis and Treatment of Leishmaniasis,” microscopic examination of bone marrow, lymph node, or splenic aspirates remains the gold standard for VL diagnosis.^23^ Notably, none of the four cases presented in the current study exhibited LD bodies on initial bone marrow smears. Two cases (B and C) required repeat bone marrow examinations to definitively identify LD bodies. One case (D) was identified via mNGS of peripheral blood, which suggested leishmaniasis, prompting targeted reevaluation of the bone marrow. The remaining case (A) was confirmed after a comprehensive review of the travel history, prompting repeated sampling and meticulous microscopic examination. The diagnostic challenges stem from the typically low parasite burden found in pediatric patients during acute presentations or intermittent disease, as well as the inherent sensitivity limitations of conventional bone marrow smear techniques. These limitations necessitate specialized expertise for accurate microscopic identification, particularly because of the morphological similarities between LD bodies, characterized by basophilic cytoplasm, prominent purple nuclei, and kinetoplasts, and PLT aggregates. Key distinguishing features are as follows: 1) PLT aggregates are generally found extracellularly, without cellular overlap, whereas LD bodies are mainly confined to cytoplasmic compartments, having been phagocytosed by macrophages, and 2) isolated LD bodies display perinuclear cytoplasm, characterized by a pale blue halo around the purple nuclei, in contrast to PLT aggregates, which appear as amorphous purple granules or clusters without accompanying cytoplasmic staining. Serological diagnostics, particularly rK39 antigen-based immunochromatographic assays, serve as valuable adjunctive tools. However, Hagos et al.^24^ demonstrated that the rK39 antigen-based immunochromatographic assay exhibited relatively lower sensitivity (88.11%) and specificity (83.33%) than alternative methods for detecting LD. Although commercially available rapid test strips, which offer operational simplicity and cost-effectiveness, have been implemented in China, clinicians must remain vigilant regarding the potential for false-negative results, particularly during serological window periods or in immunocompromised hosts who are unable to mount adequate antibody responses. This phenomenon was exemplified in Case B, which involved negative serology but a positive bone marrow parasitological confirmation, likely reflecting underlying immune compromise.

Notably, mNGS showed remarkable promise in VL diagnosis. In Case D, peripheral blood mNGS was successfully used to identify *Leishmania* genetic material, guiding subsequent morphological confirmation. This technology’s capacity for unbiased, high-throughput sequencing of microbial nucleic acids^25^ enables early, accurate, and rapid detection of any pathogenic microorganisms (bacteria, viruses, fungi, or parasites), provided their genetic material is present in the clinical specimen.^26^ Therefore, for critically ill patients with protracted and recurrent disease courses, mNGS can provide earlier and more effective diagnostic clues for clinical decision-making. Moreover, when VL is suspected, but bone marrow smears are negative for Leishman-Donovan bodies, mNGS enables rapid and accurate etiological diagnosis, thereby facilitating precision medicine interventions.

The clinical imperative for accurate diagnosis is underscored by the stark prognostic differences between HLH generally (approaching 40% mortality^27^^,^^28^) and VL-associated HLH, which typically exhibits favorable outcomes with appropriate antiparasitic therapy. Current therapeutic paradigms emphasize that most patients achieve disease control through targeted anti-*Leishmania* treatment alone, rendering immunochemotherapeutic agents (e.g., etoposide, cyclosporine) generally unnecessary.^29^^,^^30^ Similar principles are specified in the “Chinese Guidelines for Diagnosis and Treatment of Hemophagocytic Syndrome (2022 Edition),”^31^ which states that HLH associated with certain intracellular pathogens (including LD and *Brucellosis*) may achieve durable remission through pathogen-directed therapy without adjunctive immunomodulators or cytotoxic drugs. The present case series mirrored these findings. All four patients initially received HLH-directed protocols before VL diagnosis, achieving only transient symptom control with persistent underlying disease activity. After confirmation of VL and the initiation of sodium stibogluconate therapy (with concurrent tapering of antimicrobials, immunomodulators, and cytotoxic agents), all patients experienced rapid resolution of HLH without disease recurrence, ultimately meeting the criteria for PR. This therapeutic course highlights two key clinical observations: 1) the critical role of timely sodium stibogluconate administration in managing VL-associated HLH, and 2) the potential to avoid unnecessary cytotoxic therapies and their associated complications through early and accurate etiological diagnosis. Quantitative analysis revealed significant improvements (*P* <0.05) in key hematologic parameters, including WBC counts, HGB levels, PLT counts, and LDH levels, highlighting these measures as reliable biomarkers for monitoring disease progression and assessing therapeutic response in patients with VL-associated HLH.

Compared with Gagnaire et al.’s study,^16^ which revealed a 30% misdiagnosis rate in VL-associated HLH, the present study exhibited a similar diagnostic challenge, while emphasizing the critical importance of repeated diagnostic efforts. Gagnaire et al.^16^ indicated that the delayed diagnosis of VL in HLH cases was primarily due to the lack of suspicion for an infectious etiology, particularly in immunocompromised patients. This finding is consistent with the current study, as three of four patients initially received HLH-directed treatment without VL being considered a potential cause, and the diagnosis was confirmed only after repeat bone marrow aspiration or the use of mNGS. Furthermore, whereas Gagnaire et al.^16^ reported an average time to diagnosis of 18 days for their cohort, a broader range was found in the present case series, with delays ranging from 17 days to as long as 7 months, highlighting variability in diagnostic timelines. These delays may stem from the similar clinical manifestations of VL and HLH, including recurrent fever, hepatosplenomegaly, and pancytopenia, which may lead to initial misdiagnoses. The longer diagnostic intervals align with Bode et al.’s findings,^22^ which revealed that misdiagnosis and delayed treatment initiation in pediatric patients with VL-associated HLH are frequent, particularly in resource-limited settings. Moreover, treatment strategies vary across studies, with some authors advocating early initiation of anti-*Leishmania* therapy as the cornerstone of remission in HLH.^29^^,^^30^ The approach presented in the current study, aligning with the recommendations of Gagnaire et al.,^16^ prioritizes the prompt administration of sodium stibogluconate as soon as VL is suspected, leading to favorable outcomes in all cases. In contrast, patients who received HLH-directed chemotherapy without specific anti-*Leishmania* therapy exhibited only transient symptom relief, highlighting the importance of etiological diagnosis in guiding treatment options. In terms of outcomes, the present case series revealed significant improvements in laboratory parameters, consistent with findings reported in other case series. Notably, post-treatment normalization of ferritin levels, WBC counts, PLT counts, and LDH levels was noted in all patients, a key indicator of therapeutic efficacy, confirming the results of Gagnaire et al.^16^ and other contemporary studies.^19^^,^^24^ The analysis further supports that VL-associated HLH can achieve favorable outcomes when diagnosed early and treated with targeted anti-*Leishmania* therapy. However, as reported in previous studies, including those conducted by Gagnaire et al.^16^ and Bode et al.,^22^ the time to diagnosis remains challenging. The challenges associated with differentiating VL from other causes of HLH highlight the need for enhanced awareness among clinicians, especially in endemic areas. The present study further emphasizes the need for a multi-faceted diagnostic approach that incorporates travel history, clinical presentation, repeated bone marrow sampling, serological tests, and advanced molecular techniques, such as mNGS, to ensure timely diagnosis. However, the interpretation of the improvement in cytokine levels, including sCD25 and ferritin levels, as part of the therapeutic efficacy assessment was confirmed via specific comparisons in Supplemental Table 1. This allows for a clearer understanding of how each parameter contributes to the overall conclusion of PR in all patients, addressing concerns about transparency and completeness in efficacy evaluation.

The present study has several limitations that should be considered. Firstly, the small sample size, drawn exclusively from inpatients at the study institution, may not adequately represent the characteristics of patients across diverse global endemic regions. Although the findings in the current case series provide valuable insights into the diagnostic and therapeutic challenges of VL-associated HLH, the generalizability of these results is limited by the small sample size (*n* = 4) and the single-center design. Consequently, the conclusions, particularly regarding the success of de-escalating immunotherapy, should be interpreted with caution because they may not apply to broader patient populations without further validation in larger, multicenter studies. Secondly, among the cases reviewed, only Patient A returned for follow-up bone marrow reexamination to confirm parasite clearance, whereas the other three patients lacked microbiological verification of cure. The specific characteristics of this cohort, including travel histories to endemic regions and the clinical context of the study institution, further restrict the extrapolation of these findings. Finally, only Case A had microbiological confirmation of parasite clearance via follow-up bone marrow examination, which was essential in confirming a definitive cure. However, because post-treatment microbiological data are absent for the other three patients, the conclusion of a definitive “cure” for all cases is less robust. Although clinical outcomes, such as fever resolution, normalization of laboratory parameters, and improvement in hepatosplenomegaly, indicate therapeutic efficacy, the lack of follow-up bone marrow or other parasitological evidence in Cases B, C, and D limits confidence in parasite eradication. This highlights the need for consistent follow-up evaluations, including bone marrow reassessment, to definitively confirm parasite clearance in cases of VL-associated HLH. Larger studies incorporating diverse patient populations and clinical settings are required to confirm these results and develop more comprehensive guidelines for managing VL-associated HLH.

## CONCLUSION

In conclusion, the present study highlighted the diagnostic challenges and therapeutic efficacy in managing pediatric cases of VL-associated HLH. The findings revealed the critical role of mNGS in confirming VL diagnosis, especially in cases in which conventional bone marrow smear microscopy fails to identify *Leishmania* amastigotes. Notably, mNGS showed significant potential for early, accurate diagnosis, as seen in Case D, in which it enabled prompt pathogen identification and emerged as pivotal in altering the clinical course of the disease. In addition, hematological parameters, such as WBC count, HGB level, PLT count, and LDH level, were found to be reliable biomarkers for monitoring treatment response. Statistically significant improvements in these parameters post-treatment not only improved the effectiveness of sodium stibogluconate therapy in VL-associated HLH but also highlighted their utility as key indicators of clinical outcomes. Specifically, WBC count, HGB level, PLT count, and LDH level exhibited marked recovery after VL-specific treatment, confirming therapeutic benefit and aiding in clinical decision-making. Furthermore, the present study revealed the importance of a systematic diagnostic approach, particularly in endemic regions, to differentiate VL from other causes of HLH and ensure timely and appropriate interventions. Overall, the findings support integrating mNGS and key hematological parameters as essential components in the diagnostic and treatment protocols for VL-associated HLH, thereby improving patient outcomes and guiding future clinical practice in similar settings.

## Supplemental Materials

10.4269/ajtmh.25-0530 Supplemental Materials
