# Supplementary material for: Clinical Characteristics and Management of Four Cases of Visceral Leishmaniasis-Associated Hemophagocytic Lymphohistiocytosis
Source: Am J Trop Med Hyg. 2026 Mar 26;114(5):882–8. doi: 10.4269/ajtmh.25-0530 (PMC13153628; doi:10.4269/ajtmh.25-0530)
Supplement: Supplemental Materials [file tpmd250530.SD1.pdf]

Supplemental Figure 1. Phagocytosed LD bodies within macrophages in bone marrow smear of Case D (Wright-Giemsa staining ×1,000).

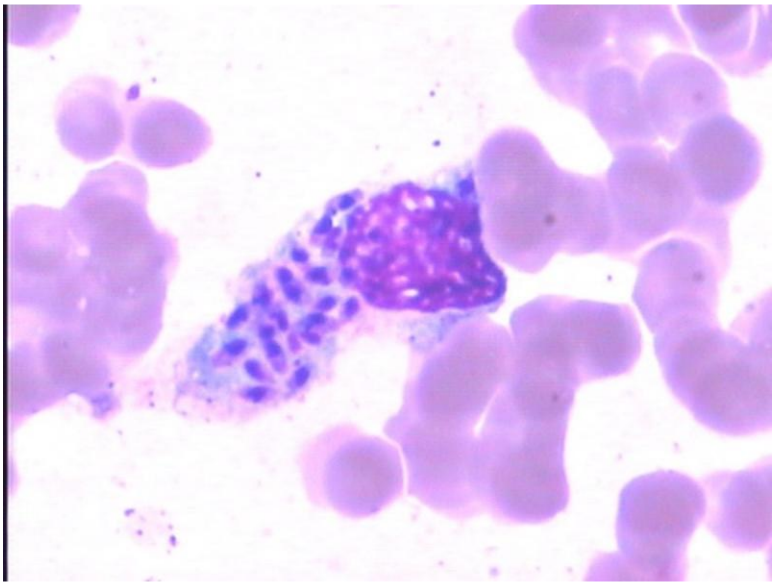

Supplemental Figure 2. Scattered extracellular LD bodies in bone marrow smear of Case D (Wright-Giemsa staining ×1,000).

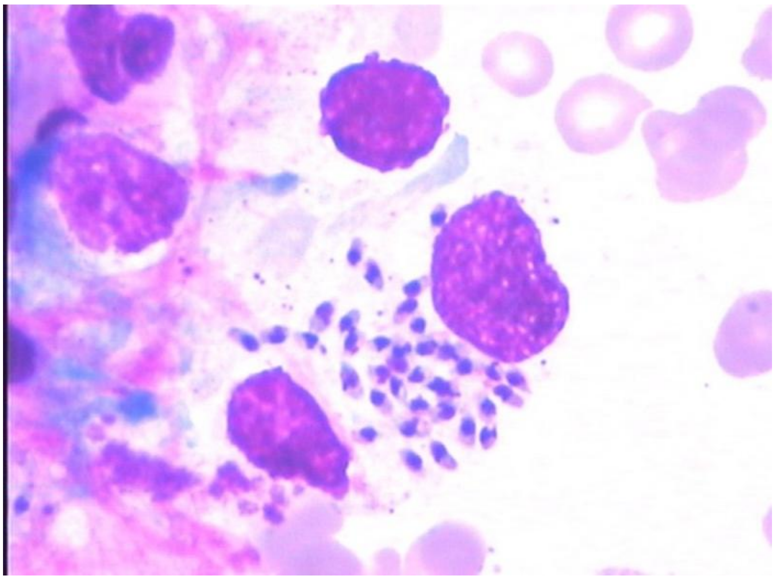

Supplemental Figure 3. Post-treatment bone marrow smear from Case A showing absence of LD bodies (Wright-Giemsa staining ×1,000).

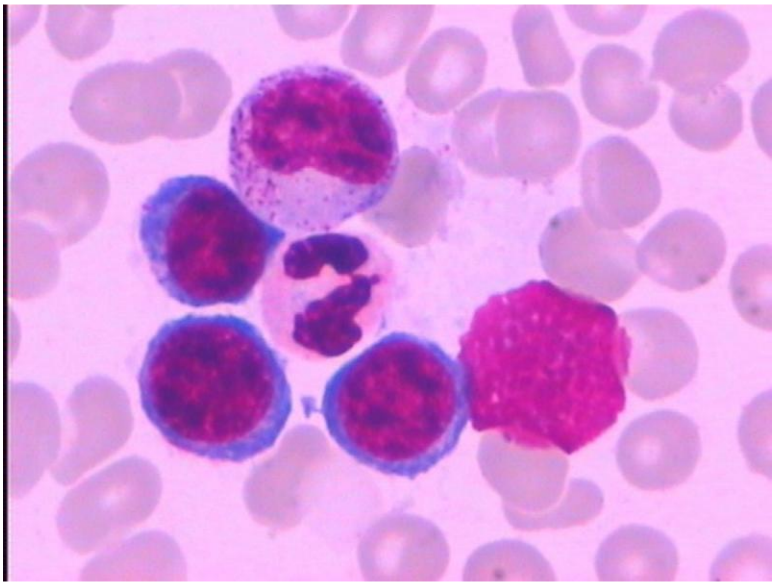

Supplemental Figure 4. Post-treatment bone marrow smear from Case A showing absence of LD bodies (Wright-Giemsa staining ×1,000).

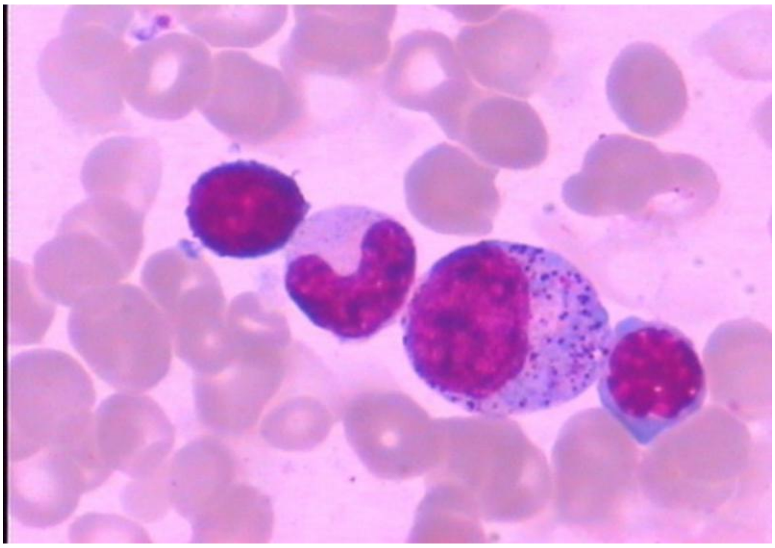

Supplementary Table 1. Laboratory parameters of the 4 patients before and after antimonial therapy

	A			B			C			D		
	Pre-treatment	Post-treatment	Efficacy evaluation	Pre-treatment	Post-treatment	Efficacy evaluation	Pre-treatment	Post-treatment	Efficacy evaluation	Pre-treatment	Post-treatment	Efficacy evaluation
WBC (10^9/L)	3.00	8.22	Returned to normal	2.44	9.33	Returned to normal	3.73	6.22	Returned to normal	1.05	4.97	Returned to normal
ANC (10^9/L)	0.47	1.11	Returned to normal	0.21	3.45	Returned to normal	1.07	1.39	Normal	0.37	1.28	Returned to normal
HGB (g/L)	73	112	Returned to normal	70	92	Increased by > 25%	85	111	Returned to normal	87	102	Returned to normal
PLT (× 10^9/L)	56	140	Returned to normal	46	300	Returned to normal	51	195	Returned to normal	185	167	Returned to normal
LDH (U/L)	864	332	Decreased by > 25%	1463	306	Decreased by > 25%	661	216	Returned to normal	702	385	Decreased by > 25%
sCD25 (U/L)	—	—	—	> 44000	Retest result in another hospital was normal	Returned to normal	> 44000	2822	Decreased by > 1/3	15316	——	——
TG (mmol/L)	2.52	0.94	Returned to normal	3.49	3.99	Increased	3.55	2.04	Increased	1.56	——	——
ALT (U/L)	51	23.9	Normal	20.9	11.6	Normal	13.3	14.6	Normal	25.7	14.9	Normal
Ferritin (ng/mL)	> 2000	1050.52	Decreased by > 1/3	> 2000	1054.12	Decreased by > 1/3	1141.98	24.23	Returned to normal	6093.13	——	——
Efficacy evaluation	PR			PR			PR			PR		

——: not tested

Supplementary Table 2. Comparison of clinical indicators in the 4 patients before and after treatment

	Before treatment	After treatment	t	*P*
WBC (10^{12}/L)	2.56±1.13	7.19±1.96	-4.940	0.016*
ANC (10^{12}/L)	0.53±0.38	1.81±1.10	-1.921	0.151
HGB (g/L)	78.75±8.5	104.25±9.32	-5.058	0.015*
PLT (10^{9}/L)	59.50±17.48	200.50±70.03	-3.479	0.040*
LDH (U/L)	922.50±370.84	309.75±70.62	3.282	0.046*
ALT (U/L)	27.73±16.33	16.25±5.31	1.957	0.145

*The difference was statistically significant (P<0.05).
